# Bartonellas: could they cause reproductive disorders in humans?

**DOI:** 10.1590/S1678-9946202567015

**Published:** 2025-03-03

**Authors:** Rafaela de Paula Silva, Marina Rovani Drummond, Paulo Eduardo Neves Ferreira Velho

**Affiliations:** 1 Universidade de Campinas, Faculdade de Ciências Médicas, Laboratório de Pesquisa Aplicada em Dermatologia e Infecções por Bartonela, Campinas, São Paulo, Brazil; 2 Universidade de Campinas, Faculdade de Ciências Médicas, Divisão de Dermatologia, Campinas, São Paulo, Brazil

Campinas, January 28^th^, 2025

Dear Editor,

Bartoneloses are millennial diseases. There are *Bartonella* species that have universal distribution, such as *Bartonella henselae*, which is the species most associated with disease in humans. Although infection by these bacteria can be fatal, as in the case of endocarditis, infected humans can also be asymptomatic, as observed in 500 blood donors from the UNICAMP Blood Bank of Campinas, Sao Paulo State, Brazil, of which more than 20% showed detection of *B. henselae* DNA or isolation of the bacteria, which reveals the high prevalence of infection among asymptomatic subjects^1^.

Although Maillard *et al*.^2^ observed no relationship between *Bartonella bovis* and *Bartonella chomelii* infection in dairy cattle herds and their offspring, different *Bartonella* species have already been associated with reproductive difficulties in mammals. Reproductive disorders such as abortion and fetal resorption have been described in female cats experimentally infected with *B. henselae* ^3^. Guptill *et al*.^4^ experimentally demonstrated that *B. henselae* causes reproductive failure in female cats, but transplacental transmission was not evidenced. A recent study demonstrated that *B. henselae* DNA was amplified from fetal and placental samples from stray cats that were frequently positive for bacterium in reproductive tissues^5^.

Another study linked the presence of *B. henselae* to the probable cause of foal abortion, and this is the first documentation of the species causing abortion in horses^6^. Kosoy *et al*.^7^ isolated *Bartonella* species in embryos and neonates of naturally infected rodents. Subsequently, the pathogenic effect on the reproductive functions of BALB/c mice experimentally infected with *Bartonella birtlesii* was described by Boulouis *et al*.^3^, who demonstrated increased fetal death and lower birth weights of viable fetuses. Transplacental transmission has been documented by isolation of the bacteria from fetuses^8^.

Siewert *et al*.^9^ recently wrote about how the adaptive immune defense prevents *Bartonella* persistence in transplacental transmission in C57BL/6 mice. Furthermore, de Bruin *et al*.^10^ reported *Bartonella schoenbuchensis* DNA in all life stages of *Lipoptena cervi* ticks, except for three fully developed pupae. The DNA was found in various types of ticks, including wingless adult males and females collected from both red deer and roe deer, as well as in larvae harvested from adult females. This reinforces the notion that *Bartonella* is transmitted vertically from wingless females to larvae that will develop into pupae.

We understand that from a One Health perspective, *Bartonella* spp. infection may be related to immunoprotection or immunoaggression via the vertical route in humans as well, as described for the animals studied by the authors. They cite vertical transmission of *Bartonella* sp. in small rodents^9^, but also in humans in the case reported by Breitschwerdt *et al*.^8^. In this report, the possible transmission of *B. henselae* and *Bartonella vinsonii* subsp. *berkhoffii* from a woman to her twin sons, conceived after *in vitro* fertilization, was found. One of the newborns died nine days after birth due to hypoplastic left heart syndrome. *Bartonella* sp. DNA was detected in the blood and/or paraffin tissue of the four family members: the woman, her husband, and the twins. The authors point out that since there were difficulties for the woman to become pregnant naturally, which persisted after consecutive *in vitro* fertilizations, the case highlights the possibility that *Bartonella* sp. infection can negatively influence human reproductive performance.

Another case reported by Velho *et al*.^11^ also reinforces the hypothesis of vertical transmission of *Bartonella* sp. in humans and describes a three-year-old child born to an asymptomatic woman, for whom the possibility of vertically transmitted *B. henselae* infection was hypothesized because the baby presented anemia, jaundice, and hepatosplenomegaly since birth. This suspicion was reinforced by transmission electron microscopy findings of the patient’s liver fragment collected during a biopsy performed at 12 days of life and molecular detection by species-specific reaction of *B. henselae* DNA directly from three blood samples from the child and a blood sample from the mother. The amplifications were sequenced and found to be 100% homologous to *B. henselae*.

The possibility of vertical transmission of *Bartonella bacilliformis* has also been reported in a newborn with acute manifestation of Carrion’s bartonellosis whose mother had skin lesions characteristic of the chronic phase of the disease^12^.

Bilavisky *et al*.^13^ described eight pregnant women diagnosed with cat scratch disease (CSD) during a 19-year case surveillance study of this disease in Israel. One of them had a miscarriage with no documented association with bartonellosis, and the others had neither obstetric complications nor did their newborns manifest clinical expressions of bartonellosis. Agarwal *et al*.^14^, on the other hand, reported a case of liver rupture with a diagnosis of *B. henselae* infection caused by CSD in a 24-year-old pregnant woman.

Thus, the study by Siewert *et al*.^9^ reminds us of the need to evaluate the role of *Bartonella* sp. infection in women with repeat abortions and the role of transplacental transmission of these bacteria in humans in the same way as in mice, cats and horses.
